# Health services availability and readiness moderate cash transfer impacts on health insurance enrolment: evidence from the LEAP 1000 cash transfer program in Ghana

**DOI:** 10.1186/s12913-022-07964-w

**Published:** 2022-05-04

**Authors:** Peter Otieno, Gustavo Angeles, Sarah Quiñones, Vincent van Halsema, Jacob Novignon, Tia Palermo, the LEAP 1000 Evaluation Team, Tia Palermo, Richard de Groot, Elsa Valli, Isaac Osei-Akoto, Clement Adamba, Joseph K. Darko, Robert Darko Osei, Francis Dompae, Nana Yaw, Sudhanshu Handa, Clare Barrington, Sara Abdoulayi, Gustavo Angeles, Averi Chakrabarti, Frank Otchere, Akalpa J. Akaligaung, Raymond Aborigo

**Affiliations:** 1 African Population and Health Research Center, P.O. Box 10787-00100, Nairobi, Kenya; 2 Department of Maternal and Child Health, UNC Gillings School of Global Public Health, 400 Meadowmont Circle CB #3446, Chapel Hill, NC USA; 3 Department of Epidemiology and Environmental Health, University at Buffalo, SUNY, 270 Farber Hall, Buffalo, NY USA; 4 UNICEF Ghana, P.O. Box 5051, 4-8th Rangoon Close, Accra, Ghana; 5 Department of Economics, Kwame Nkrumah University of Science and Technology, Kumasi, Ghana

**Keywords:** Social protection, Health insurance, Supply-side, Health services, Ghana

## Abstract

**Background:**

Expanding health insurance coverage is a priority under Sustainable Development Goal 3. To address the intersection between poverty and health and remove cost barriers, the government of Ghana established the National Health Insurance Scheme (NHIS). Government further linked NHIS with the Livelihood Empowerment Against Poverty (LEAP) 1000 cash transfer program by waiving premium fees for LEAP 1000 households. This linkage led to increased NHIS enrolment, however, large enrolment gaps remained. One potential reason for failure to enroll may relate to the poor quality of health services.

**Methods:**

We examine whether LEAP 1000 impacts on NHIS enrolment were moderated by health facilities’ service availability and readiness.

**Results:**

We find that adults in areas with the highest service availability and readiness are 18 percentage points more likely to enroll in NHIS because of LEAP 1000, compared to program effects of only 9 percentage points in low service availability and readiness areas. Similar differences were seen for enrolment among children (20 v. 0 percentage points) and women of reproductive age (25 v. 10 percentage points).

**Conclusions:**

We find compelling evidence that supply-side factors relating to service readiness and availability boost positive impacts of a cash transfer program on NHIS enrolment. Our work suggests that demand-side interventions coupled with supply-side strengthening may facilitate greater population-level benefits down the line. In the quest for expanding financial protection towards accelerating the achievement of universal health coverage, policymakers in Ghana should prioritize the integration of efforts to simultaneously address demand- and supply-side factors.

**Trial registration:**

This study is registered in the International Initiative for Impact Evaluation’s (3ie) Registry for International Development Impact Evaluations (RIDIE-STUDY-ID-55942496d53af).

**Supplementary Information:**

The online version contains supplementary material available at 10.1186/s12913-022-07964-w.

## Background

Expanding health insurance coverage is a target under Sustainable Development Goal 3 (promoting good health and well-being for all) [1]. While the global focus on financial protection has increased over the past decade [2], persistent gaps in the breadth of coverage remain [3, 4]. In Sub-Saharan Africa (SSA), the vast majority of individuals still rely on direct out-of-pocket payments (OOPs) for healthcare services despite the renewed commitments by most national governments toward universal health coverage (UHC) [5, 6]. Every year, millions of individuals in SSA are pushed into abject poverty as a result of catastrophic health expenditures [6]. Such barriers in access to essential services contribute to high burdens of preventable mortality [7]. Given the rising demand for affordable quality health care services and low utilization rates of essential services, several countries in SSA have implemented healthcare financing reforms to mitigate financial barriers among vulnerable populations [5].

In Ghana specifically, despite expanded social health protection in the past two decades, the country has continued to report poor health outcomes across several domains in the 21^st^ century. The 2020 under-5 mortality rate was 46 per 1000 live births against the 2030 SDG target of 25 per 1000 live births [8, 9]. And, the proportion of deliveries by skilled health personnel stands at 71% against the SDG target of 90% [10]. Ghana is currently experiencing a rapid epidemiological transition with the double burden of infectious diseases and non-communicable diseases (NCDs). The crude mortality rate stands at 7.2 per 1,000 people as of 2019 [11]. Results of a recent systematic review and meta-analysis suggest that more than a quarter of the adult population are currently living with hypertension [12]. However, only 22% of those with hypertension are on treatment [12]. NCDs account for about 43% of all deaths. Moreover, Ghana was ranked among 15 countries with the highest burden of malaria in the world in 2019 and malaria remains a leading cause of death followed closely by lower respiratory infections, ischemic heart diseases, HIV/AIDS, and Tuberculosis [13, 14].

Over the past two decades, Ghana has implemented several policy initiatives to address the ravaging impact of poverty on the health of its citizens, as well as the reinforcing nature of poor health and poverty. Two of these initiatives are the National Health Insurance Scheme (NHIS) and Livelihood Empowerment Against Poverty (LEAP) program, and both are described in more detail below.

### Financing healthcare in Ghana

In Ghana, there exists a combination of public and private payers for health insurance. The NHIS is the largest payer for health insurance in the country, while there also exist several private health insurance payers. The National Health Insurance Authority NHIA) is mandated to manage the NHIS and also regulate private payers including issuance and renewal of operation licences. The NHIA purchases services from both private and public providers who are accredited by the NHIA. In this way, subscribers can seek care from both private and public providers without incurring additional costs.

The NHIS was established in 2003 through Act 650 of parliament and was later amended as Act 852 in 2012. The implementation of the scheme began in 2004 to remove user fees at the point of care [15–17]. The scheme is part of the poverty reduction strategy in Ghana and aims to ensure equitable access to essential quality healthcare services for all. The NHIS aims to remove cost barriers to accessing care and covers about 95% of all diseases and/or conditions in the country. Specific conditions covered under the scheme include malaria, acute respiratory tract infection, diarrhoeal disease, skin disease, and ulcers, hypertension, acute eye infection, rheumatism, anaemia, intestinal worms disorders, acute ear infection, typhoid fever, dental caries, diabetes mellitus, STIs, asthma, laboratory services, ultrasound scans and x-rays, HIV/AIDS symptomatic treatment for opportunistic infections, out-patient/day surgical operations, out-patient physiotherapy, prescription medicines on the National Health Insurance Scheme Medicines List, and traditional medicines approved by the Food and Drugs Board and prescribed by accredited medical and traditional medicine practitioners [18]. The scheme also covers oral health, eye care services, maternity care, and emergency medical services (including brain or heart surgery due to accidents, paediatric emergencies, obstetric and gynaecological emergencies, road traffic accidents, and industrial and workplace accidents) [18].

Membership subscription to the NHIS is legally mandatory for all formal employees through the Social Security and National Insurance Trust (SSNIT) contributions [19]. Enrolment in the NHIS is, however, voluntary for informal sector workers and formal sector workers who do not contribute to SSNIT [19]. Such workers voluntarily pay the NHIS registration/processing fees and premiums to get coverage [19]. However, there are exemption strategies that waive premiums and/or processing fees for specific population groups. Act 852 originally exempted the following groups from paying the NHIS premium as well as processing fees: persons classified as poor or indigent, LEAP beneficiaries, persons in need of antenatal, delivery, and postnatal healthcare services, persons with mental disorders, and persons categorized as disabled and determined to need social welfare support. The following group of people are exempted from paying premiums but do pay processing fees: children under 18 years, persons over seventy years, contributors to the Social Security and National Insurance Trust (SSNIT), pensioners of the SSNIT. For those exempt from premium payments, registration and renewal fees are 8 and 5 Ghana Cedis (GH₵; 0.76 and 1.22 USD), respectively. For the non-exempt group, premiums are based on income levels ranging from 7.2 to 48 GH₵ (1.1-7.3 USD^i^). However, in the absence of data to judge income levels, flat rates are charged, ranging from 15 to 22 GH₵ (2.3-3.4 USD^i^), where urban dwellers are expected to pay relatively more than those in rural areas [20].

Premium levels for NHIS were originally managed at the district level by district mutual health insurance schemes (DMHIS) and approved by the NHIA. However, as of 2012, the management of premiums has become centralized. The scheme is mainly financed by the National Health Insurance Fund (NHIF) levy, contributions from social security and premium payments from members, allocations by parliament, donor support, returns on investments by the NHIA council, and contributions by employees in the formal sector through SSNIT [21]. The scheme is heavily subsidized by the government.

While the active membership (i.e., current possession of a valid NHIS card) of NHIS has significantly expanded over the past decade, there are wide gaps in enrolment [22]. Approximately 40% of the Ghanaian population is currently enrolled in the NHIS and about two-thirds currently fall into a premium exempt category, with the indigent group constituting 14% of NHIS members [23]. Almost half (48%) of members are pregnant women and children under 18 years of age [23].

### The Ghana LEAP program

The Ghana LEAP Program was implemented in 2008 by the Ministry of Gender, Children and Social Protection (MoGCSP) to address extreme poverty. The program provides bimonthly cash payments (ranging from 64 GH₵ for households with one member to 106 GH₵ for households with four or more members; 10-17 USD) to extremely poor households with orphans and vulnerable children, elderly with no productive capacity, persons with severe disability, and, starting in 2015, those with a pregnant woman or child under the age of 12 months [24, 25]. LEAP reached more than 213,000 extremely poor families in all districts across Ghana as of December 2017 (the year in which data were last collected for the current study) [26] and has continued to be scaled up, with an enrolment of 350,000 households (covering 1.5 million individuals) by the end of 2021. To integrate social protection programming with the health sector, MoGCSP worked with the National Health Insurance Authority (NHIA) in 2011 and secured LEAP participating households fee waivers to enroll in the NHIS under the NHIA “indigent” exemption category [24]. Thus, the LEAP program is comprised of a bi-monthly cash transfer and a premium fee waiver for enrolment into NHIS.

When LEAP eligibility criteria were expanded to include poor households with a pregnant woman or child under the age of 12 months, this expansion was first piloted in 10 districts and the pilot was called “LEAP 1000.” The Upper East and Northern Regions were selected for this expansion based on criteria of high proportions of people in poverty (74.8 and 60.2% living in the lowest wealth quintile, respectively [27]) and incidence of poor nutrition (prevalence of stunting is 14.4 and 33.1% for children under the age of 5, respectively [28]). The eligibility criteria expansion was in recognition of the fact that the LEAP program was not previously reaching many households with young children, including many suffering from malnutrition. To reduce malnutrition and stunting rates, it was recognized that the first 1,000 days of life is a critical period of development with long-term implications for health and wellbeing [24]. The aims of LEAP 1000 were to reduce stunting and improve the welfare of young children in poor households in Ghana, and it was believed that targeting pregnant women in extremely poor households was a key point of entry to achieve these aims.

The efforts of the Government of Ghana to integrate anti-poverty social protection programming in the form of LEAP with social health protection in the form of NHIS recognize that social protection programs’ target populations are often overlapping but that explicit efforts are needed to better integrate programs to ensure that participants are accessing all services to which they are entitled. This integration can also better address the multidimensional nature of poverty.

An evaluation of LEAP 1000 found that the program was successful at achieving many of its aims, including increasing overall consumption, reducing the poverty headcount and gap, improving household-level food insecurity [26, 29], and increasing enrollment in NHIS (Palermo et al., 2019). In terms of secondary objectives, the program was successful in increasing antenatal care seeking, exclusive breastfeeding, and pre-school enrollment. There were also positive benefits beyond program objectives, whereby LEAP 1000 reduced intimate partner violence [26, 30] and increased support among women [31]. Nevertheless, the program was not successful at reducing stunting or improving other nutritional outcomes among children [26].

Some challenges could limit the effectiveness of LEAP and NHIS integration. For instance, despite the fact that LEAP 1000 increased the probability of NHIS enrolment by 15 percentage points, fewer than half of eligible LEAP participants enrolled in NHIS even though they qualified for a fee waiver [32]. Drivers of this gap in insurance uptake include limited knowledge of the scheme and supply-side limitations including drug and staff shortages as well as long wait times [23, 32, 33]. This suggests that there are other important drivers of health insurance enrolment other than the cost of annual premiums [34], and while the aforementioned study by Palermo and colleagues examined the extent to which LEAP 1000 increased NHIS enrolment [32], it did not consider whether program impacts varied based on contextual factors. For example, it is possible that households in areas with greater service availability and readiness and better health infrastructure might be more responsive to fee waivers because they can more easily envision the benefits of health care services.

### Barriers to health insurance uptake

Previous studies have shown that factors and characteristics such as lack of understanding of the concept of health insurance, low perceived service quality, long distances and reduced accessibility to services, low trust in the scheme insurer, younger age, unmarried status, and male gender, [35], as well as travel costs and lost wages for the trip needed to renew enrolment each year [36], may lower the uptake of health insurance. Other studies have identified perceived low returns to enrolment among individuals who consider themselves to be relatively healthy [37–39].

Another set of studies have demonstrated how contextual factors such as the quality of services influences health services utilization. For example, these studies have examined the effects of the health workforce and medical equipment availability and probability of seeking care in Mozambique [40], availability of essential medicines and household healthcare utilization in Tanzania [41], perceived service quality, and behavioral intentions to seek healthcare in Ghana [42], quality of services and sick child health services utilization in Malawi [43], services readiness and the likelihood of facility delivery in Haiti [44], family planning services quality and contraceptive use in Egypt, Morocco, and Tanzania [45–47], and service provision characteristics and antenatal care in Zambia [48].

However, no study has examined how the effects of a cash transfer combined with a premium fee waiver on health insurance enrolment might be moderated by supply-side factors such as the quality and availability of health services. In the current study, we examine whether impacts of an integrated social protection program, comprised of a cash transfer combined with a fee waiver for NHIS enrolment, on NHIS enrolment were moderated by health facilities’ service availability and readiness in Ghana. This study builds on previous evidence demonstrating that the intervention examined did increase health insurance uptake [32], but that study did not examine the moderating influence of supply-side factors, which has important implications for scale-up and future program design, including information about what complementary programming is required.

## Methods

### Setting

This study was conducted in five districts in the Northern part of Ghana (Yendi, Karaga, East Mamprusi in the Northern Region, and Bongo and Garu Tempane^1^ in the Upper East Region). Demographic and geographic information for each of these five districts is detailed in Supplementary Table 1. Agriculture is the predominant sector in these regions, and households from the study areas are largely engaged in subsistence agriculture and petty trade.

### Program targeting and enrolment

LEAP 1000 participating households were targeted for enrolment by the government between March and July 2015. The following were required for proof of eligibility: a) antenatal cards (if pregnant); or b) birth certificates and weighing cards among households with infants less than 15 months. Those who applied for the program were then administered a proxy means test (PMT), which assessed assets, dwelling characteristics, household size, and related characteristics, and assigned a score to determine poverty status. Households meeting the poverty criterion were enrolled starting in August 2015. A total of 6,124 poor households with pregnant women and infants were enrolled into LEAP 1000 in 2015. The distribution of LEAP 1000 eligible households is presented in Supplementary Table 1. The LEAP 1000 pilot program, targeting, and entitlements are detailed elsewhere [24, 26].

### Data and study design

A quasi-experimental, longitudinal impact evaluation was implemented by UNICEF Office of Research – Innocenti, the University of North Carolina at Chapel Hill (UNC-CH), the Institute of Statistical, Social and Economic Research (ISSER) of the University of Ghana, and Navrongo Health Research Center (NHRC) to assess the impacts of LEAP 1000. The evaluation covered five of the original ten LEAP 1000 pilot districts, which were chosen to reflect the demographic diversity of the pilot areas. This impact evaluation used government targeting data for the sampling frame and sampled households around the eligibility cut-off.

Data for the current study come from the baseline (2015, prior to enrolment in LEAP 1000) and end-line (2017) rounds of the impact evaluation. The evaluation uses a regression discontinuity design (RDD)-inspired approach to identify a comparison group, exploiting the program eligibility score (PMT) used in the targeting phase. This RDD design focuses on observations near the cut-off (on both sides), and this approach is sometimes referred to as local randomization [49]. The following assumptions must hold for RDD to be valid: 1) the threshold for program eligibility should be exogenous (it was determined by the government after PMT data was collected and based on budget availability); 2) the distribution of the score around the cut-off should not show any discontinuity at baseline (discontinuities would indicate manipulation of scores by participants to qualify for the program); 3) distribution of household characteristics and outcomes should not show any discontinuity at the cut-off point and should be statistically balanced. These assumptions were found to hold in previous analyses [24].

Households with scores below the PMT cut-off were classified as extremely poor and enrolled in LEAP 1000, while those with scores above the cut-off were not eligible. Those who were close to the cut-off but did not qualify were used as the comparison group for the impact evaluation. At baseline (July-September 2015), a total of 2,497 households (1,235 comparison and 1,262 treatment) were interviewed across five districts and 93.4% of the total baseline sample were interviewed again at end-line (June and August 2017; 92.8% comparison and 93.9% treatment). Household questionnaires were administered to gather information on household composition; education and health of household members; enrolment in NHIS; housing conditions and water, sanitation, and hygiene (WASH); food security; time use, and productive activities; among other outcomes. At baseline, health facility surveys were administered to staff in 142 health facilities (see Supplementary Fig. 1) in the targeted districts. We assume little change to health facility characteristics from baseline to end-line.

### Availability of data and materials

The data used in this analysis are publicly available from the University of North Carolina Population Center (https://data.cpc.unc.edu/projects/13/view#res_226).

### Ethics review and study registration

The original evaluation study was reviewed by the Ethics Committee for the Humanities of the University of Ghana. The trial is registered in the International Initiative for Impact Evaluation’s (3ie) Registry for International Development Impact Evaluations (RIDIE-STUDY-ID-55942496d53af). The current analysis uses de-identified data and was exempted from IRB review at the University at Buffalo.

### Measures

The primary outcome examined in this study was current NHIS enrolment, defined at the individual-level. Enrolment was assessed via a series of questions administered to one household survey respondent about all household members aged five years and above. Questions included whether the individual was covered under any health insurance scheme, and possible responses to this question included “National/District Health Insurance (NHIS)”, “Mutual Health Organization/Community-based Health Insurance”, “Other Privately Purchased Commercial Health Insurance”, or “Other Health Insurance.” Respondents were then asked if the individual currently had a valid NHIS card, used to validate current enrolment status.

To assess health facility quality, we used data from health facility surveys conducted as part of the original impact evaluation and created a health facility service availability and readiness scale, based on the World Health Organization Service Availability and Readiness Assessment (WHO SARA) guidelines to the extent that data were available [50]. SARA captures information on service delivery (including service availability) and the readiness of health facilities to provide basic care to patients. Dimensions assessed under this index include amenities, basic equipment, infection prevention, diagnostic capacity, and essential medicines. Each indicator was coded as =1 if available and =0 otherwise, and then means were calculated to create the service availability index (ranges from 0-1). Readiness indicators include power, adequate sanitation facilities, communication equipment, emergency transportation, and more, as well as services-specific indicators on guidelines, checklists, trained staff, equipment, and drugs. We developed sub-scales for basic amenities, basic equipment, diagnostic capacity, and essential medicines. Additionally, we developed service availability and readiness sub-scales for family planning, antenatal care services, immunization, and child health services. By averaging all sub-scales, a general service quality (availability and readiness) scale was developed for each facility. Supplementary Table 2 provides an overview of the SARA indicators that were used in this analysis to construct health facility quality scales. Tertiles of the final scales were calculated to classify health facilities as having low, moderate, or high health quality (Supplementary Fig. 1). Health facility indicators were then linked to sample households using GPS coordinates via spatial join in ArcMap 10.7.1.

### Analysis

We first summarize enrolment status by background characteristics. Next, LEAP 1000 impacts on NHIS enrolment were estimated with Difference-In-Differences (DD) estimation methods, comparing changes in enrolment between baseline and end-line for the treatment group with changes over the same time period in the comparison group. We use a triple differences (DDD) model to examine the moderating impacts of SARA tertiles. The estimating equation is as follows:

1 $${E}_{ijt}={\beta }_{0}+{\beta }_{1}{P}_{ij}+{\beta }_{2}{T}_{t}+{\beta }_{3}{SARAT2}_{ij}+{\beta }_{4}{SARAT3}_{ij}+{\beta }_{5}\left({P}_{ij}*{T}_{t}\right)+{\beta }_{6}\left({P}_{ij}*{SARAT2}_{ij}\right)+{\beta }_{7}\left({P}_{ij}*{SARAT3}_{ij}\right)+{\beta }_{8}\left({T}_{t}*{SARAT2}_{ij}\right)+{\beta }_{9}\left({T}_{t}*{SARAT3}_{ij}\right)+{\beta }_{10}\left({P}_{ij}*{T}_{t}*{SARAT2}_{ij}\right)+{\beta }_{11}\left({P}_{ij}*{T}_{t}*{SARAT3}_{ij}\right)+X\beta +{\varepsilon }_{ijt}$$

NHIS enrolment is indicated by ${E}_{ijt}$ for individual i in community j at time t. LEAP 1000 program participation is represented by P. Survey rounds are indicated with T and it takes the value of 1 for endline and 0 for baseline, SARAT2 is equal to 1 if the individual resides in a community linked to a facility classified as SARA tertile 2, and SARAT3 takes the value of 1 if the individual resides in a community linked to a facility classified as SARA tertile 3. X is a vector of baseline control variables (PMT score, household size, whether the household head was female, head’s age in years, and a dummy variable indicating whether the household head had no education), and $\varepsilon$ is the error term. The coefficient of the interaction term on program participation and time ${\beta }_{5}$ indicates the intent-to-treat program impact among those in the lowest SARA tertile (the reference group). Program impacts on individuals residing in communities where health facilities are classified in the middle SARA tertile is represented by the combination of the following coefficients: $({\beta }_{5}+{\beta }_{10})$. Program impacts on individuals residing in communities where health facilities are the highest SARA tertile is represented by the combination of the following coefficients: $({\beta }_{5}+{\beta }_{11})$. To determine program impacts for these second and third tertiles, we estimated the joint significance of these coefficients using the *lincom* command in Stata. We ran models stratified by age (7-17 years and 18 years and up at endline) to take into consideration varying health care needs across the life-course, as well as age-related targeting of the LEAP 1000 program. We also ran a separate model for women of reproductive age (defined as 15-49 years). All models adjust standard errors for clustering at the community level.

## Results

### Background characteristics

Table 1 shows background characteristics by NHIS enrolment status. Individuals less likely to have current NHIS enrolment (among both children and adults) include those in larger households and those living in Garu Tempane, Karaga, and Yendi districts compared to living in Bongo district. Children (but not adults) in households where the household head did not have a formal education and those living in a household headed by older persons were also less likely to be enrolled in NHIS. Among adults (but not children), females and individuals living in a female-headed household were more likely to be enrolled in NHIS. Among women of reproductive age, women in larger households, those in households where the head lacked formal education, and those living in Garu, Karaga, and Yendi districts compared to living in Bongo district were less likely to be enrolled. There were no differences in enrolment by poverty status.

**Table 1:** Bivariable analyses of background characteristics by enrolment status

	% Current NHIS enrolment					
	Ages 7-17 years at end-line			Ages 18+years at end-line		
	No	Yes	*P*-values	No	Yes	*P*-values
Sex						
Male (Ref)	53.7	53.2		51.2	28.9	
Female	46.3	46.9	0.60	48.8	71.1	0.00
Household size, *M(SD)*	8.4(3.1)	8.0(3.0)	0.00	7.3(3.0)	7.2(2.9)	0.04
Sex of household head						
Males (Ref)	93.8	92.7		94.7	92.6	
Female	6.3	7.3	0.25	5.3	7.4	0.00
Age of head, *M(SD)*	42.7(11.3)	41.6(11.2)	0.00	39.7(12.4)	40.0(13.1)	0.85
Formal schooling of head						
Yes (Ref)	13.5	18.1		19.3	21.0	
No	86.5	81.9	0.00	80.7	79.0	0.06
Poor						
No (Ref)	4.5	5.3		6.3	7.2	
Yes	95.5	94.7	0.30	93.7	92.8	0.19
Extreme poverty						
No (Ref)	19.5	21.8		23	24.4	
Yes	80.5	78.2	0.10	77	75.6	0.25
Bongo District (Ref)	9.8	16.3		15.6	18.7	
East Mamprusi District	28.1	38	0.29	28.2	37.8	0.46
Garu-Tempane District	17.7	12.4	0.00	13.1	11	0.00
Karaga District	23.9	19.2	0.00	28	19.3	0.00
Yendi District	20.4	14.2	0.00	15.2	13.2	0.01
N	4,867	3,507		8,126	4,092	

Source: Authors’ analysis. *p*-values correspond to the significance levels of estimated coefficients for current NHIS enrolment in a linear regression where the outcome is the characteristic in each row, and models additionally control for PMT score.Standard errors are clustered at the community level

*M* Mean, *NHIS* National Health Insurance Scheme, *PMT* Proxy means test, *SD* Standard deviation, *Ref* Reference category

Data presented as column % unless otherwise specified

### Characteristics of healthcare facilities

**Fig. 1:**
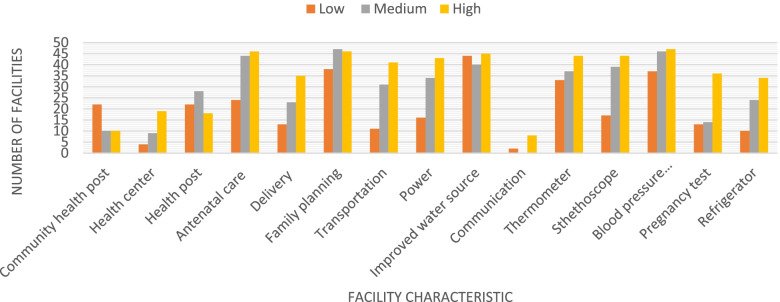
Health facility characteristics by tertile of service quality

Table 2 summarizes the characteristics of health facilities examined in this study. The largest number of facilities (*n*=50) are found in the Bongo district, and Yendi had the fewest (*n*=12). The distribution of types of facilities in the study are community health posts (29.58%), health centers (22.53%), and health posts (47.89%). Among these facilities, 80.28% provide antenatal care, while only 50% provide delivery services. While 90.85% have an improved water source, only 65.49% have a regular source of power. In terms of basic equipment, 91.55% have a blood pressure apparatus, 70.42% have a stethoscope, and 47.89% have a refrigerator. When examining these characteristics across the service availability and readiness tertiles, we see large differences. For example, only 33% of facilities in the first tertile have power, while 91% in the third tertile do (Fig. 1 and Supplementary Table 3). Other differences are seen comparing across the first and third tertiles for the availability of transportation (23% v. 87%), stethoscope (35% v. 94%), availability of HIV tests (29% v. 77%), contraceptive impacts (67% v. 98%), and antenatal care services (50% v. 98%), among others. Supplementary Table 3 also shows that 15% of low-tertile health facilities carried IUDs compared to 6% of middle-tertile facilities while 43% of middle-tertile facilities carried fansidar compared to 38% of high-tertile facilities.

**Table 2:** Health facilities characteristics

	Overall (*N*=142)	
**Characteristic**	**N**	**Mean**
District		
Bongo	50	35.21
East Mamprusi	13	9.15
Garu Tampane	52	36.62
Karaga	15	10.56
Yendi	12	8.45
Facility Type		
Community health post	42	29.58
Health centre	32	22.53
Health post	68	47.89
Services		
Antenatal care	114	80.28
Delivery	71	50
Family planning	131	92.25
Amenities		
Transportation	83	58.45
Power	93	65.49
Improved water source	129	90.85
Communication	10	7.04
Equipment		
Thermometer	114	80.28
Stethoscope	100	70.42
Blood pressure apparatus	130	91.55
Pregnancy test	63	44.37
Refrigerator	68	47.89

### Moderating impact of service quality on NHIS uptake

Table 3 shows the impacts of LEAP 1000 on NHIS uptake, moderated by service availability and readiness (ranked into tertiles). Among children (Column 1), there were no impacts of LEAP 1000 on NHIS enrolment in the lowest service availability and readiness tertile, but among the middle and highest tertiles, LEAP 1000 increased enrolment by 13 and 20 percentage points, respectively. Among adults (Column 2), LEAP 1000 leads to a 9-percentage point increase in NHIS enrolment in areas with the lowest service availability and readiness. In areas with middle and highest service availability and readiness (tertiles 2 and 3), LEAP 1000 led to a 15 and 18-percentage point increase, respectively, in NHIS enrolment. Turning to women of reproductive age (Column 3), we find that LEAP 1000 increased NHIS enrolment by 10 percentage points in areas with the lowest service availability and readiness, while the program increased NHIS enrolment by 11 and 25 percentage points among women in areas with the middle and highest service availability and readiness, respectively.

**Table 3:** Moderating impact of service availability and readiness on LEAP 1000 impacts on NHIS enrolment

	Ages 7-17 years at end-line	Ages 18+years at end-line	Women of reproductive age (15-49 years)
DD (Treatment*time)	0.08	0.09	0.10
	(0.06)	(0.04) **	(0.06) *
Treatment	-0.02	0.01	0.03
	(0.05)	(0.03)	(0.04)
Time	-0.08	0.00	-0.08
	(0.05)	(0.03)	(0.04) **
Service quality readiness tertiles			
Lowest (Ref)			
Middle	-0.06	-0.09	-0.09
	(0.07)	(0.04)**	(0.06)
Highest	0.00	0.00	0.03
	(0.07)	(0.04)	(0.06)
Moderating impact of service quality readiness tertiles			
(a) Treatment			
Lowest*Treatment (Ref)			
Middle*Treatment	0.03	0.02	0.04
	(0.06)	(0.04)	(0.05)
Highest*Treatment	-0.05	-0.05	-0.11
	(0.06)	(0.04)	(0.04) **
(b) Time			
Lowest*Time(Ref)			
Middle*Time	-0.04	-0.02	0.03
	(0.06)	(0.04)	(0.05)
Highest*Time	-0.12	-0.13	-0.13
	(0.09)	(0.05) **	(0.06) **
(c) DDD			
Lowest*Treatment*Time (Ref)			
Middle*Treatment*Time	0.05	0.07	0.01
	(0.08)	(0.05)	(0.07)
Highest*Treatment*Time	0.12	0.10	0.15
	(0.08)	(0.05) *	(0.07) **
PMT score	-0.18	0.02	0.03
	(0.21)	(0.16)	(0.18)
Household size	-0.01	0.00	-0.01
	(0.00) ***	(0.00) *	(0.00) ***
Head is female	0.01	0.07	0.02
	(0.03)	(0.02) **	(0.03)
Age of head	0.00	0.00	0.00
	(0.00)	(0.00)	(0.00)
Head no formal schooling	-0.07	-0.03	-0.03
	(0.03) **	(0.02) *	(0.02)
N	8,374	12,218	5,910
Total impact – low SARA tertile	0.08 (0.06)	0.09 (0.04)**	0.10 (0.06)*
Total impact – middle SARA tertile	0.13 (0.05)***	0.15 (0.03)***	0.11 (0.04)***
Total impact – highest SARA tertile	0.20 (0.06)***	0.18 (0.03)***	0.25 (0.05)***

*DID* Difference-in-difference, *NHIS* National Health Insurance Scheme, *PMT* Proxy means test, *Ref* Reference category

Source: Authors’ analysis; All regressions include the following covariates at baseline: age, dummy for female (0,1), household head’s age, dummy for having no formal education (0,1), dummy for women household head (0,1), PMT score, household size

Impact from DID estimates; impact on ever NHIS enrolment from single difference estimates. Analysis restricted to a panel sample. Robust standard errors in parentheses clustered at the community level. Total impacts on middle and highest quality tertiles estimated with Lincom command in Stata

^*^p<0.1, **p<0.05, ***p<0.01

## Discussion

We examined whether impacts of the LEAP 1000 cash transfer program in Ghana on NHIS enrolment were moderated by nearby health facilities’ service readiness and availability. This is the first study to examine how the quality of services moderates a cash transfer’s impact on health insurance uptake. Our findings demonstrate large moderating effects of service availability and readiness on LEAP 1000’s ability to increase NHIS enrolment (a 9-percentage point difference in LEAP 1000 impacts on enrolment between adults residing in areas with the lowest and highest tertiles of service availability and readiness and a 15-percentage point difference among women of reproductive age).

Our findings confirmed our hypothesis that high service quality amplified the impacts of LEAP 1000 on NHIS enrolment among all groups examined (children, adults, and women of reproductive age). The largest moderating impacts were seen among children (followed by women of reproductive age) living in high-quality areas versus those living in low-quality areas. This suggests that contextual factors can have large influences on cash transfer impacts based on lifecycle characteristics of the targeted population. For example, women of reproductive age might be more sensitive to contextual factors due to their high relative demand for health services, including those related to antenatal care and delivery.

A previous study among LEAP 1000 beneficiaries found that the primary reasons for non-enrolment in NHIS despite eligibility for premium fee waivers were perceptions of the high cost of premiums, costs of travel to renew the card, and lack of understanding that NHIS enrolment expires and must be renewed annually [32]. These findings suggest the need for better communication with participants who are eligible for premium fee waivers. It is also possible that individuals in areas where service availability and readiness were higher interacted with more knowledgeable health professionals who were more likely to communicate to LEAP 1000 participants their eligibility for NHIS premium fee waivers; however, we cannot test this hypothesis with our available data.

We found evidence of significant variation in service readiness and availability across the study areas. Consistent with our findings, previous studies have also shown marked heterogeneity of service quality across healthcare facilities in Ghana characterized by disparities in the supply of essential medicines, medical equipment, and other critical healthcare resources [51, 52]. Despite the establishment of the National Healthcare Quality Strategy in 2016, a study conducted in 2017 by the WHO revealed widespread quality implementation challenges [53]. Likewise, the Institutional Care Division mandated to develop and implement clinical quality standards in Ghana has also found critical gaps in service availability and readiness [54]. Other findings from earlier studies in Ghana point to a primary focus on the demand side of the healthcare services [55, 56]. However, the ripple effect of the rise in the demand for healthcare services is an increase in the economic stress on the supply side with perceived negative impacts on the service quality [57].

Our findings are consistent with two existing bodies of literature. The first body of literature indicates that service availability and readiness influence health-related behaviors. For example, healthcare utilization was associated with health service availabilityin Mozambique [40], and continuous availability of essential medicines in Tanzania [41]. Further, improved health facility and structural quality were associated higher odds of child healthcare services in Malawi [43] and in Haiti, greater health facility delivery service availability and readiness were associated with an increase in facility deliveries [44]. In Ghana, a qualitative study found that barriers to NHIS enrolment included inadequate service availability given sparsely distributed health facilities, poor NHIS administration, and perceived poor quality of care [42].

The second body of literature is more limited but demonstrates that contextual factors can moderate the impacts of cash transfers. For example, in Zambia, quality of health services were found to moderate impacts of the Child Grant Program on skilled birth attendance [58]. Impacts of the same program on stunting and height-for-age z-scores were also moderated by access to clean water [59]. Additionally, impacts of the Kenya Cash Transfer for Orphans and Vulnerable Children on school enrolment and success were moderated by proximity to and cost of schools [60]. Findings from these studies support our findings that contextual factors are considerable moderators of cash transfer impacts.

Also consistent with our results on moderating impacts of quality, the 2014 Ghana Demographic and Health Survey revealed a significant association between perceived quality of healthcare services and NHIS enrolment [61]. Another study found that poor service quality led to a drop out of health insurance enrolment in Ghana [62]. Other studies have also linked the expansion of health insurance schemes to increased healthcare utilization and upward pressure on available services that may negatively impact the quality of service and, subsequently, willingness to subscribe to health insurance [63, 64]. The findings of our study in concert with other studies suggest 1) health service quality is an important contributor to health insurance uptake; 2) interventions that improve demand-side factors increase health insurance enrolment; and 3) enhancing supply-side factors will augment the impacts of demand-side improvements on health insurance enrolment in this context.

### Strengths and limitations

Our study has several strengths. This is the first study to examine how the effects of a demand-side intervention aimed at increasing health insurance enrolment is moderated by supply-side characteristics. Second, we use a quasi-experimental, longitudinal study design to examine whether the causal impacts of the intervention were moderated by health facilities’ service readiness and availability. Third, the assessment of the quality of healthcare is based on an objective evaluation of a wide range of indicators comprising of basic amenities, equipment, diagnostic capacity, essential medicine, and availability of maternal and child health services. Lastly, we use spatial join maps and GPS coordinates to link primary care facilities to the nearest households.

This study has some limitations. First, the assessment of health facility quality was based on the service availability and readiness of the respondent's nearest public health facility. It is possible that the respondents may have bypassed the nearest healthcare facilities in favor of other distant facilities, due, in part, to patient experiences, cost of healthcare, and other factors [65, 66]. However, a recent nationally representative survey on the tendency to bypass the nearest health facility in Ghana revealed that the vast majority of Ghanaians (over two-thirds) sought care from their nearest facility [67]. Thus, the assumptions made in our analyses are unlikely to introduce a large bias. Second, the service availability and readiness indicator was constructed without taking into account the client perceived perspective. We acknowledge the importance of the perceptions of clients [68]. However, previous studies have revealed a higher likelihood of bias from client perceived quality than objective assessments due to the tendency by the clients to respond favorably to quality indicators [69–71]. Therefore, we believe that the service availability and readiness indicator used in our study (and recommended by WHO) is a useful measure. Additionally, our facility survey did not include private health facilities, where NHIS card holders may seek services if the facility has been accredited by NHIA. Lastly, the study design implemented estimates local average treatment effect. The moderating effect of healthcare quality on the impact of LEAP 1000 may be larger for individuals in poorer households, who are further from the proxy means test cut-off used in our sampling criteria and thus not included in our study, compared with households who are relatively better off (but still poor) and included in our study.

Our results highlight the need for further policy discourse and studies on best practices of how to integrate efforts towards service quality improvement with those aimed at increased NHIS coverage. Such policy shifts may not only have the potential to improve health insurance coverage but also form part of long-term strategies for driving universal health coverage in Ghana. A focus on the demand-side factors without equal consideration of the supply-side factors may result in a vicious cycle characterized by inadequate access to quality healthcare services, discouraging enrolment of new members [5]. Thus, interventions targeted at improving service availability and readiness may be a key to improving health insurance coverage in Ghana. It is worth mentioning that there have been recent efforts to improve service availability across the country, including efforts to construct 111 district health facilities across the country. While this is a step in the right direction, it is important to ensure the structures are stocked with the needed resources to enhance availability and readiness. In addition to constructing health facilities, efforts to retain rural health workers as well as attract private health sector investors to rural (or deprived) areas will be important steps to improve health service quality [72, 73]. More research is also needed on how service availability and readiness can affect other population health outcomes in addition to NHIS enrolment.

## Conclusions

We find compelling evidence that supply-side factors relating to service readiness and availability boost positive impacts of a cash transfer program on NHIS enrolment. Our work suggests that demand-side interventions coupled with supply-side strengthening may facilitate greater population-level benefits down the line. In the quest for expanding financial protection towards accelerating the achievement of universal health coverage, policymakers in Ghana should prioritize the integration of efforts to simultaneously address demand- and supply-side factors.

## Supplementary Information

**Additional file 1: Supplementary Figure 1.** Map of Health facilities in study areas, by SARA tertile, Ghana LEAP 1000 Evaluation

**Additional file 2: Supplementary Table 1.** Population and Area of each District included in the LEAP 1000 impact evaluation

**Additional file 3: Supplementary Table 2.** Indicators used for SARA scales development

**Additional file 4: Supplementary Table 3.** Summary of SARA items by tertile of service readiness

## Data Availability

The dataset supporting the conclusions of this article is publicly available at the University of North Carolina Population Center (https://www.data.cpc.unc.edu/projects/13/view#res_226).

## Footnotes

1 Garu Tempane became two separate districts: Garu and Tempane in 2018. At the time of the Ghana LEAP 1000 pilot (2015-2017), this remained a single district.
