# Supplementary material for: Health services availability and readiness moderate cash transfer impacts on health insurance enrolment: evidence from the LEAP 1000 cash transfer program in Ghana
Source: BMC Health Serv Res. 2022 May 4;22:599. doi: 10.1186/s12913-022-07964-w (PMC9066897; doi:10.1186/s12913-022-07964-w)

**Supplementary Figure 1. Map of Health facilities in study areas, by SARA tertile, Ghana LEAP 1000 Evaluation**


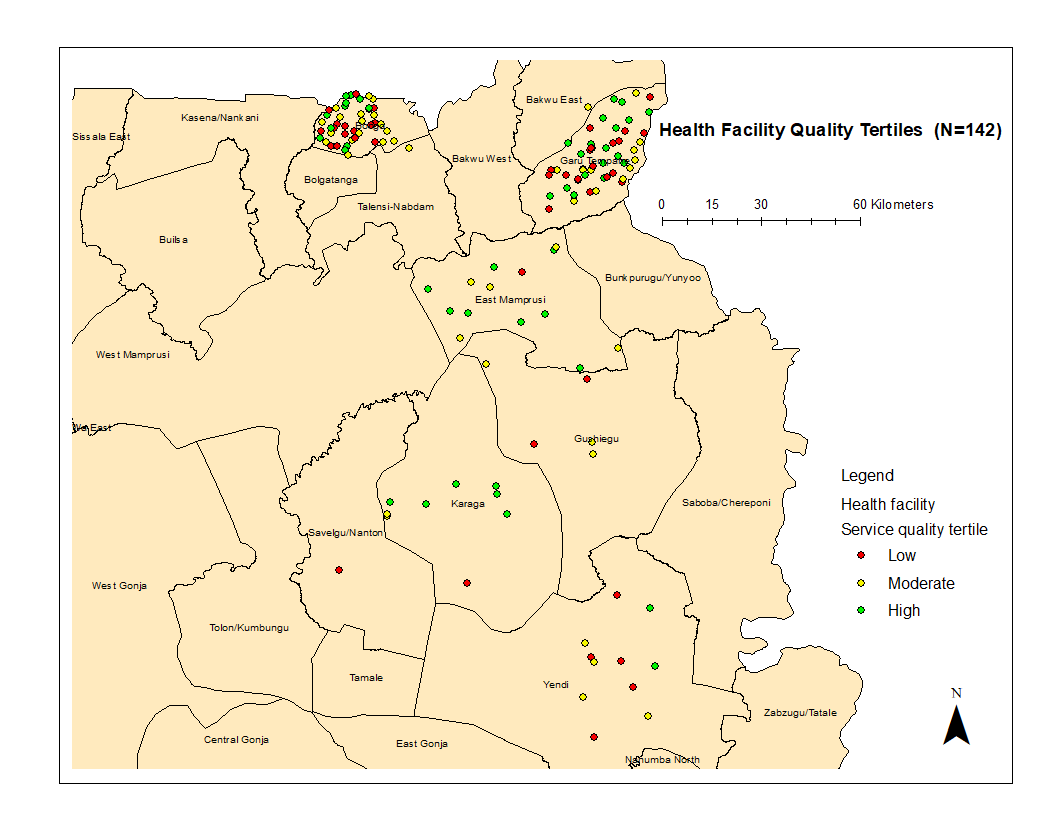

Supplement: Supplementary file 1 — Additional file 1: Supplementary Figure 1. Map of Health facilities in study areas, by SARA tertile, Ghana LEAP 1000 Evaluation [file 12913_2022_7964_MOESM1_ESM.docx]
