# Supplementary material for: Health services availability and readiness moderate cash transfer impacts on health insurance enrolment: evidence from the LEAP 1000 cash transfer program in Ghana
Source: BMC Health Serv Res. 2022 May 4;22:599. doi: 10.1186/s12913-022-07964-w (PMC9066897; doi:10.1186/s12913-022-07964-w)
Supplement: Supplementary file 2 — Additional file 2: Supplementary Table 1. Population and Area of each District included in the LEAP 1000 impact evaluation [file 12913_2022_7964_MOESM2_ESM.docx]

| **District** | **Population (2021)** | **Area** | **Population Density (2021)** | **Annual Population Change (2010-2021)** | **LEAP 1000 sample** |
| --- | --- | --- | --- | --- | --- |
| East Mamprusi | 188,006 | 1,776 km^2^ | 105.9 per km^2^ | 4.2% | 810 |
| Karaga | 114,225 | 2,758 km^2^ | 41.42 per km^2^ | 3.6% | 483 |
| Yendi | 154,421 | 1,529 km^2^ | 101.0 per km^2^ | 2.5% | 388 |
| Bongo | 120,254 | 414.9 km^2^ | 289.8 per km^2^ | 3.3% | 419 |
| Garu | 71,774 | 659.8 km^2^ | 108.8 per km^2^ | 1.9% | 397 |
| Tempane | 86,993 | 401.5 km^2^ | 216.7 per km^2^ | 1.8% |  |

**Supplementary Table 1**

**Population and Area of each District included in the LEAP 1000 impact evaluation**

Source: <https://www.citypopulation.de/en/ghana/admin/>

Source: [LEAP 1000 Baseline Report_final.pdf](file:///C:\Users\squin\Dropbox\Ghana%20LEAP%201000%20(UNC-OOR)\Baseline%20Report\Final\LEAP%201000%20Baseline%20Report_final.pdf)

Garu Tempane became two separate districts: Garu and Tempane in 2018. At the time of the Ghana LEAP 1000 pilot (2015-2017), this remained a single district.
