# Supplementary material for: Health services availability and readiness moderate cash transfer impacts on health insurance enrolment: evidence from the LEAP 1000 cash transfer program in Ghana
Source: BMC Health Serv Res. 2022 May 4;22:599. doi: 10.1186/s12913-022-07964-w (PMC9066897; doi:10.1186/s12913-022-07964-w)
Supplement: Supplementary file 3 — Additional file 3: Supplementary Table 2. Indicators used for SARA scales development [file 12913_2022_7964_MOESM3_ESM.docx]

**Supplementary Table 2. Indicators used for SARA scales development**

| **Domain** | **Tracer indicator** | **Indicators included** |
| --- | --- | --- |
| **Basic amenities** | Power | Electricity & generator |
|  | Improved water source | Public tap, borehole, protected well – no information on location of water source |
|  | Room with visual and auditory privacy | Unavailable |
|  | Adequate sanitation facilities | Unavailable |
|  | Communication equipment | Functioning landline telephone |
|  | Computer and internet access | Unavailable |
|  | Emergency transportation | Any vehicle that facility has |
| **Basic equipment** | Adult scale | Has functioning adult scale |
|  | Child scale | Has functioning child scale |
|  | Thermometer | Has clinical thermometer |
|  | Stethoscope | Has stethoscope(s) |
|  | Blood pressure apparatus | Has blood pressure apparatus |
|  | Light source | Unavailable |
| **Standard precautions for infection prevention** | Safe final disposal of sharps | Unavailable |
|  | Safe final disposal of infectious wastes | Unavailable |
|  | Appropriate storage of sharps waste | Unavailable |
|  | Appropriate storage of infectious waste | Unavailable |
|  | Disinfectant | Unavailable |
|  | Single use – standard or auto-disable syringes | Uses single-use syringes |
|  | Soap and running water or alcohol based hand rub | Unavailable |
|  | Latex gloves | Has latex gloves |
|  | Guidelines for standard precautions | Unavailable |
| **Diagnostic capacity** | Haemoglobin | Unavailable |
|  | Blood glucose | Unavailable |
|  | Malaria diagnostic capacity | Conducts rapid diagnostic tests or Malaria Parasite Screening (MPS) |
|  | Urine dipstick – protein | Urine test |
|  | Urine dipstick – glucose | Urine test |
|  | HIV diagnostic capacity | HIV tests |
|  | Syphilis rapid test | Rapid plasma reagin (RPR) test |
|  | Urine test for pregnancy | Pregnancy test |
| **Essential medicines** | Amlodipine tablet or alternative calcium channel blocker | Unavailable |
|  | Amoxicillin syrup/suspension or dispersible tablet | Unavailable |
|  | Amoxicillin tablet | Unavailable |
|  | Ampicillin powder for injection | Unavailable |
|  | Aspirin cap/tab | Aspirin |
|  | Beclometasone inhaler | Unavailable |
|  | Beta blocker (e.g.bisoprolol, metoprolol, carvedilol, atenolol) | Unavailable |
|  | Carbamazepine tablet | Unavailable |
|  | Ceftriaxone injection | Unavailable |
|  | Diazepam injection | Unavailable |
|  | Enalapril tablet or alternative ACE inhibitor e.g. lisinopril, ramipril, perindopril | Unavailable |
|  | Fluoxetine tablet | Unavailable |
|  | Gentamicin injection | Unavailable |
|  | Glibenclamide tablet | Unavailable |
|  | Haloperidol tablet | Unavailable |
|  | Insulin regular injection | Unavailable |
|  | Magnesium sulphate injectable | Unavailable |
|  | Metformin tablet | Unavailable |
|  | Omeprazole tablet or alternative such as pantoprazole, rabeprazole | Unavailable |
|  | Oral rehydration solution | Oral rehydration salt |
|  | Oxytocin injection | Unavailable |
|  | Salbutamol inhaler | Unavailable |
|  | Simvastatin tablet or other statin e.g. atorvastatin, pravastatin, fluvastatin | Unavailable |
|  | Thiazide (e.g. hydrochlorothiazide) | Unavailable |
|  | Zinc sulphate tablets, dispersible tablets or syrup | Unavailable |
| **Family planning** | | |
| **Service availability** | Family planning services | Offers family planning services |
|  | Provision of combined oral contraceptive pills | Carries contraceptive pills |
|  | Provision of progestin-only contraceptive pills | Carries contraceptive pills |
|  | Provision of combined injectable contraceptives | Carries injectable contraceptives |
|  | Provisions of male condoms | Carries condoms |
|  | Provision of female condoms | Carries condoms |
|  | Provision of intrauterine contraceptive device | Carries intra-uterine devices |
|  | Provision of implant | Carries contraceptive implants |
|  | Provision of cycle beads | Unavailable |
|  | Provision of emergency contraceptive pills | Unavailable |
|  | Male sterilization | Unavailable |
|  | Female sterilization | Unavailable |
| **Service readiness** | Guidelines on family planning | Unavailable |
|  | Family planning check-lists and/or job aids | Unavailable |
|  | Staff trained in family planning | Unavailable |
|  | Blood pressure apparatus | Has blood pressure apparatus |
|  | Combined estrogen progesterone oral contraceptive pills | Carries contraceptive pills |
|  | Progestin-only contraceptive pills | Carries contraceptive pills |
|  | Injectable contraceptives | Carries injectable contraceptives |
|  | Condoms | Carries condoms |
| **Antenatal care services** | | |
| **Service availability** | Antenatal care services | Offers ante-natal clinics |
|  | Iron supplementation | Carries iron tablets for pregnant women |
|  | Folic acid supplementation | Carries folic acid tablets |
|  | Intermittent Preventative Treatment in pregnancy (IPTp) for malaria | Carries Fansidar |
|  | Tetanus toxoid vaccination | Carries tetanus injection |
|  | Monitoring for hypertensive disorder of pregnancy | Unavailable |
| **Service readiness** | Guidelines on ANC | Unavailable |
|  | ANC check-lists and/or job-aids | Unavailable |
|  | Staff trained in ANC | Unavailable |
|  | Blood pressure apparatus | Has blood pressure apparatus |
|  | Haemoglobin | Unavailable |
|  | Urine dipstick – protein | Performs urine test |
|  | Iron tablets | Carries iron tablets for pregnant women |
|  | Folic acid tablets | Carries folic acid tablets |
|  | Tetanus toxoid vaccine | Carries tetanus injection |
|  | IPT drug (Sulfadoxine + Pyrimethamine(SP)) | Carries Fansidar |
|  | ITNs | Carries IT mosquito bed nets |
| **Skipped basic obstetric and newborn care & comprehensive obstetric care – dataset has very few indicators for scale development** | | |
| **Immunization** | | |
| **Service availability – SKIPPED** |  |  |
| **Service readiness** | Guidelines for child immunization | Unavailable |
|  | Staff trained in child immunization | Unavailable |
|  | Cold box/vaccine carrier with ice packs | Unavailable |
|  | Refrigerator | Has working refrigerator |
|  | Sharps container/safety box | Unavailable |
|  | Auto-disable syringes | Unavailable |
|  | Temperature monitoring device in refrigerator | Unavailable |
|  | Adequate refrigerator temperature | Unavailable |
|  | Immunization cards | Unavailable |
|  | Immunization tally sheets | Unavailable |
|  | Measles vaccine | Carries measles injection |
|  | DPT-Hib+HepB vaccine | Carries DPT injection |
|  | Oral polio vaccine | Unavailable |
|  | Pneumococcal vaccine | Carries meningitis injection |
|  | Rotavirus vaccine | Unavailable |
|  | IPV (Inactivated Poliovirus Vaccine) | Carries polio injection |
|  | HPV (Human Papillomavirus) | Unavailable |
|  | Cold chain minimum requirements | Unavailable |
|  | Energy source and power supply for vaccine refrigerator | Facility has electricity and/or generator |
|  | Types of power used for cold chain refrigeration | Facility has electricity and/or generator |
| **Child health services: preventative and curative care** | | |
| **Service availability** | Preventative and curative care for children under 5 | Unavailable |
|  | Malnutrition diagnosis and treatment | Offer treatment for acute malnutrition for children |
|  | Vitamin A supplementation | Carries vitamin A droplets |
|  | Iron supplementation | Unavailable |
|  | ORS and zinc supplementation | Unavailable |
|  | Growth monitoring | Has height and weight measurement equipment for under 5 year olds |
|  | Treatment of pneumonia | Unavailable |
|  | Administration of amoxicillin for the treatment of pneumonia in children | Unavailable |
|  | Treatment of malaria in children | Unavailable |
| **Service readiness** | Guidelines for IMCI | Unavailable |
|  | Guidelines for growth monitoring | Unavailable |
|  | Staff trained in IMCI | Unavailable |
|  | Staff trained in growth monitoring | Unavailable |
|  | Child and infant scale | Has weighing equipment for under five year olds |
|  | Length/height measuring equipment | Has height measurement equipment for under five year olds |
|  | Thermometer | Has clinical thermometer |
|  | Stethoscope | Has stethoscope(s) |
|  | Growth chart | Unavailable |
|  | Haemoglobin | Unavailable |
|  | Test parasite in stool | Performs stool tests |
|  | Malaria diagnostic test | Performs blood test for malaria (MPS and RDT) |
|  | Oral rehydration solution packet | Carries oral rehydration salt |
|  | Amoxicillin | Unavailable |
|  | Co-trimoxazole syrup/suspension | Carries cotrimoxazole |
|  | Paracetamol syrup/suspension | Carries paracetamol |
|  | Vitamin A capsules | Carries vitamin A droplet |
|  | Me-/albendazole cap/tab | Unavailable |
|  | Zinc sulphate tablets, dispersible tablets or syrup | Unavailable |

**Stock outs are not considered in the development of our health facility quality scales.**
