# Supplementary material for: Health services availability and readiness moderate cash transfer impacts on health insurance enrolment: evidence from the LEAP 1000 cash transfer program in Ghana
Source: BMC Health Serv Res. 2022 May 4;22:599. doi: 10.1186/s12913-022-07964-w (PMC9066897; doi:10.1186/s12913-022-07964-w)
Supplement: Supplementary file 4 — Additional file 4: Supplementary Table 3. Summary of SARA items by tertile of service readiness [file 12913_2022_7964_MOESM4_ESM.docx]

**Supplementary Table 3**. **Summary of SARA items by tertile of service readiness**

|  | Tertile 1 | Tertile 2 | Tertile 3 | p-value |
| --- | --- | --- | --- | --- |
| Power | 0.33 | 0.72 | 0.91 | <0.001 |
| Water | 0.92 | 0.85 | 0.96 | 0.196 |
| Communication | 0.04 | 0.00 | 0.17 | 0.003 |
| Transportation | 0.23 | 0.66 | 0.87 | <0.001 |
| Adult scale | 0.81 | 0.98 | 1.00 | <0.001 |
| Thermometer | 0.69 | 0.79 | 0.94 | 0.009 |
| Stethoscope | 0.35 | 0.83 | 0.94 | <0.001 |
| Blood pressure apparatus | 0.77 | 0.98 | 1.00 | <0.001 |
| Malaria test | 0.33 | 0.36 | 0.81 | <0.001 |
| Urine test | 0.04 | 0.00 | 0.23 | <0.001 |
| HIV test | 0.29 | 0.34 | 0.77 | <0.001 |
| Syphilis test | 0.00 | 0.00 | 0.04 | 0.129 |
| Pregnancy test | 0.27 | 0.30 | 0.77 | <0.001 |
| Refrigerator | 0.21 | 0.51 | 0.72 | <0.001 |
| Measles vaccine | 0.83 | 0.98 | 1.00 | 0.001 |
| DPT vaccine | 0.85 | 0.98 | 1.00 | 0.004 |
| BCG vaccine | 0.83 | 0.96 | 0.96 | 0.042 |
| Pneumococcal vaccine | 0.48 | 0.64 | 0.74 | 0.027 |
| Inactive polio vaccine | 0.81 | 0.98 | 1.00 | <0.001 |
| Child scale | 0.94 | 0.94 | 0.98 | 0.555 |
| Length/height measurement equipment | 0.52 | 0.72 | 0.83 | 0.004 |
| Rehydration salts | 0.92 | 1.00 | 1.00 | 0.018 |
| Co-trimoxoazole syrup/suspension | 0.42 | 0.62 | 0.77 | 0.002 |
| Paracetamol | 0.92 | 1.00 | 1.00 | 0.018 |
| Vitamin A droplets | 0.94 | 1.00 | 1.00 | 0.050 |
| Family planning services | 0.79 | 1.00 | 0.98 | <0.001 |
| Antenatal care services | 0.50 | 0.94 | 0.98 | <0.001 |
| Iron supplementation | 0.73 | 0.96 | 0.96 | <0.001 |
| Folic acid supplementation | 0.75 | 0.96 | 1.00 | <0.001 |
| Offers IPTp (fansidar) for malaria | 0.17 | 0.43 | 0.38 | 0.015 |
| Offers tetanus toxoid vaccination | 0.88 | 0.98 | 1.00 | 0.011 |
| Facility has insecticide treated mosquito nets (itns) | 0.83 | 0.98 | 0.98 | 0.006 |
| Oral contraceptives | 0.77 | 0.98 | 0.96 | 0.001 |
| Injectable contraceptives | 0.75 | 0.98 | 0.98 | <0.001 |
| Condoms | 0.79 | 1.00 | 1.00 | <0.001 |
| Contraceptive implants | 0.67 | 0.79 | 0.98 | <0.001 |
| IUD | 0.15 | 0.06 | 0.26 | 0.036 |
| *N* | 48 | 47 | 47 |  |
